# A Case of Nephrotomy

**Published:** 1880-04

**Authors:** 


### A CASE OF NEPHROTOMY.

At Guy's Hospital Mr. Clement Lucas recently removed a suppurating kidney from a man aged thirty six years. The lumbar incision was adopted and antiseptic precautions were employed. At last accounts the patient was considered almost free from danger. The man had had a purulent discharge through the loin for six years.—*Medical Record.*

